# The Early and Late Postoperative Complications of Pediatric Neuromuscular Scoliosis at King Abdulaziz Medical City, Riyadh, Saudi Arabia: A Case Series

**DOI:** 10.7759/cureus.28154

**Published:** 2022-08-19

**Authors:** Hadeel A Ashour, Ghadi a Almohaisen, Samira A Hawsawi, Maha A Aljrayed, Shihanah M AlKhelaiwi, Samir Alsayegh, Sami I Aleissa, Wael A Alshaya

**Affiliations:** 1 Medicine and Surgery, College of Medicine, King Saud bin Abdulaziz University for Health Sciences, Riyadh, SAU; 2 Pediatric Surgery, College of Medicine, King Saud bin Abdulaziz University for Health Sciences, Riyadh, SAU; 3 Orthopaedics, King Abdulaziz Medical City, Riyadh, SAU; 4 Pediatric Surgery, King Abdulaziz Medical City, Riyadh, SAU

**Keywords:** blood loss, intraoperative, complications, postoperative, neuromuscular scoliosis

## Abstract

Background

Neuromuscular Scoliosis (NMS) is defined as “a coronal plane spinal curvature of 10 degrees or more, measured by the Cobb method, in the setting of muscle imbalance secondary to an underlying neuropathic or myopathic disease”.^ ^Patients who have the disease usually manifest with diminished balance, asymmetrical seating, abnormal gait, and decreased pulmonary function, which are related to the change in spine posture. Surgery benefits patients with NMS in terms of stopping disease advancement and improving quality of life, but is known to be associated with certain complications in this population. The aim of this study is to identify the most common complication in NMS patients after surgical correction.

Methods

This study is a chart review-based retrospective case series that has covered patients’ data going from 2015 to 2019. The study focused on patients who underwent scoliosis correction surgery of both genders and mainly of a single ethnicity, with the inclusion of patients aged 9 to 18 years old. Under consecutive sampling, the study has met a sample size of 14 patients.

Results

Most of the study subjects nine (64%) were female. The age median was 13 years (2.25). The highest documented intraoperative complication was blood loss in 11 (79%) patients. The most prevalent early postoperative complication was urinary tract infection in two (14%) patients. No late postoperative complications were documented in the study.

Conclusion

The study concluded that blood loss was the most common intraoperative complication. Pulmonary problems were one of the least reported complications. Possible reasons for these findings and prevention methods should be the focus of future studies.

## Introduction

Neuromuscular Scoliosis (NMS) is defined as “a coronal plane spinal curvature of 10 degrees or more, measured by the Cobb method, in the setting of muscle imbalance secondary to an underlying neuropathic or myopathic disease” [1]. Al-Arjani et al. found that NMS is the third (11%) most common type of scoliosis in Saudi Arabia in a study composed of 192 cases of scoliosis [2]. NMS is a common spinal deformity affecting pediatric patients, negatively impacting them physiologically, physically, psychologically, and socially [3]. Since the disease manifests at a critical age in a child's social development, poor body image is one of the many issues these children might face [3]. Diminished balance, asymmetrical seating, and abnormal gait are all symptoms attributed to the change in spine posture [4]. The initial findings of physical examination are noticeable asymmetry in the buttocks, hips, and waistline, and protruding ribs on one side [4]. Once scoliosis is suspected, it is confirmed with an X-ray which is the primary diagnostic tool for confirming all types of scoliosis [5]. Regarding the treatment of NMS, there are two options: surgical and nonsurgical management [5]. Nonsurgical treatment such as physical therapy improves the quality of life without stopping disease progression [5]. On the other hand, surgical management improves the quality of life and prevents disease advancement [5].

Due to the nature of their neuromuscular disorder, these individuals have decreased pulmonary vital capacity [6]. In addition, the spinal deformity further affects the ability of their lungs to fully expand during inspiration [6]. Most NMS patients are non-ambulatory which negatively impacts their respiratory function and the outcome of the correctional surgery [7]. All of the mentioned factors along with the long duration of the surgery can influence the frequency and severity of postoperative complications [8]. Studies conducted with regard to the complications of correctional surgery of NMS have showcased similar complication rates, especially pulmonary complications [9-12]. One retrospective study conducted by Luhmann and Furdock found that the highest postoperative complications were pulmonary complications with a rate of 15.3% among 111 NMS patients [9]. Another study done by Matsumoto et al. revealed that after correctional surgery for NMS cases, pulmonary and cardiovascular complications required intensive care [10]. Mohamad et al. conducted a study composed of 175 NMS patients who underwent correctional surgery, which showed a total perioperative complication rate of 33.1%, 19.4% of which were pulmonary complications [11]. Some of the most common respiratory consequences were pneumonia, pleural effusion, pneumothorax, and prolonged tracheal cannula duration [9]. In Saudi Arabia, one study done by Almalki et al. investigated the outcomes of scoliosis surgery and reported one case of neurological complications and one case of wound infection among 82 cases [13].

As of this date, there are only a few studies conducted in Saudi Arabia concerning the postoperative complications amongst NMS patients. The aim of this study is to identify the most common complication during and after spinal surgical correction of pediatric NMS patients in King Abdulaziz Medical City (KAMC), Riyadh, Saudi Arabia.

## Materials and methods

This study is a retrospective case series that has covered patients’ data going from 2015 to 2019. King Abdullah International Medical Research Center issued approval for this study with an approval number SP20/268/R. We picked KAMC as our setting since it is a tertiary care hospital, it has numerous specialized care centers serving national guard soldiers and their families, and also patients from all regions of Riyadh. The inclusion criteria were patients who underwent correctional surgery for neuromuscular scoliosis, of both genders, mainly of a single ethnicity, aged between 9 and 18 years old, and had a follow-up period of at least two years. The exclusion criteria were patients who had growing rods (devices used to correct scoliosis while permitting the child’s spine to grow) or had another type of scoliosis than the neuromuscular type. Under consecutive sampling, the study has met 14 subjects who were operated on by multiple pediatric orthopedic surgeons. The data was collected using a data sheet to extract the information from the digital files taken from the BESTCare system (a health information system) at KAMC. The sheet included patients’ demographic characteristics such as age and gender, preoperative features such as type of neuromuscular disorder, and postoperative complications such as blood loss. The postoperative complications were divided into two periods: early postoperative and late postoperative. The early postoperative period is the first six weeks after the surgery. The late postoperative period is after the first six weeks of the surgery. Each patient's MRN was linked to a unique serial number to ensure confidentiality. Microsoft Excel was used for data entry and arrangement of the variables of interest to our study and then analyzed using the software JMP version 16 (SAS Institute Inc., Cary, NC). All categorical variables such as the presence of complications were described in frequency and percentage and all numerical variables such as age and BMI were described in median and interquartile ranges.

## Results

From the 14 cases reviewed, nine (64%) were females. The age median was 13 years (2.25) with a minimum of nine years and a maximum of 15 years. The remaining demographic characteristics are shown in Table 1.

**Table 1:** Demographic Characteristics of Patients Sample BMI = body mass index.

Sample characteristics	Frequency (N)	Percentage (%)	Median	Interquartile range (Q3-Q1)
Gender				
Female	9	64.29%		
Male	5	35.71%		
Age(years)			13	2.25
Height(cm)			128	16.75
Weight(kg)			31.15	22.21
BMI			19.37	9.85
Diagnosis				
Cerebral Palsy	8	57.14%		
Spinal Muscular Atrophy 1	3	21.43%		
Spinal Muscular Atrophy 2	1	7.14%		
Duchenne Muscular Dystrophy	2	14.29%		

**Figure 1:**
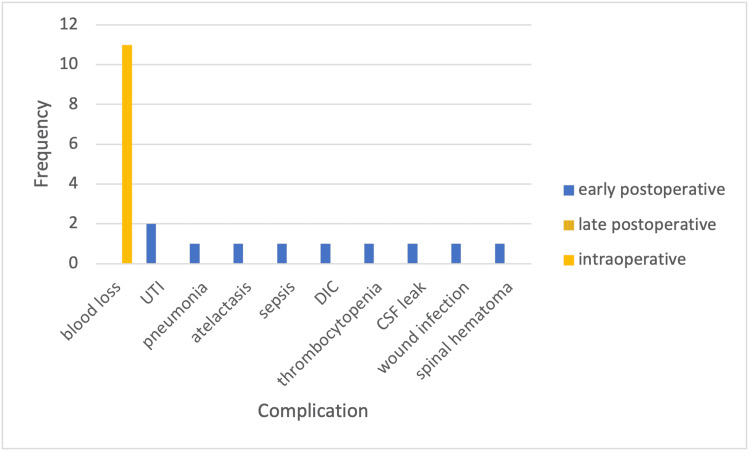
Frequency of Intraoperative, Early Postoperative, and Late Postoperative Complications UTI = urinary tract infection; DIC = disseminated intravascular coagulation; CSF = cerebrospinal fluid. The intraoperative period is during the surgery. The early postoperative period is the first six weeks after surgery. The late postoperative period is after the first six weeks of surgery.

Complications were divided into three periods: intraoperative, early postoperative, and late postoperative. The highest documented intraoperative complication was blood loss in 11 (79%) patients. The most documented early postoperative complication was urinary tract infection in two (14%) patients. The additional complications are shown in Figure 1.

Half of the patients had an estimated blood loss of less than or equal to 500ml. Only those who have received packed red blood cells during the surgery were considered complicated cases. The other postoperative characteristics are seen in Table 2. 

**Table 2:** Postoperative Characteristics of the Sample PRBCs = packed red blood cells. Extubation means the removal of the endotracheal tube. Pedicle screws were used in all patients. The level of instrumentation refers to the level at which the vertebrae were fixed using pedicle screws.

Sample characteristics	Frequency(N)	Percentage(%)	Median	Interquartile range(Q3-Q1)
Length of hospital stay (days)			9	7.5
Duration of surgery (hours)			8	1
Day of foley catheter removal			4	3.5
Day of extubation			1	1
Use of surgical drain	3	21.43%		
Number of pedicle screws used			25	7
Level of instrumentation				
T2-L5	1	7.14%		
T2-S2	12	85.71%		
T3-L4	1	7.14%		
Estimated Blood loss ≤ 500ml	7	50%		
Estimated Blood loss >500ml and ≤1000ml	4	28.57%		
Estimated Blood loss >1000ml and ≤1500ml	1	7.14%		
Estimated Blood loss >1500ml and ≤2000ml	2	14.29%		
Received PRBCs	11	78.57%		

## Discussion

Scoliosis correction surgery is a procedure associated with high risks as reported by numerous studies; pulmonary complications being the most common [9-12]. Patients suffering from a neuromuscular disorder e.g. cerebral palsy, with a scoliosis deformity are at higher risk for developing pulmonary complications such as pneumonia, atelectasis, pneumothorax, and respiratory failure [6]. Their reduced respiratory capacity is not only due to the restrictive ventilatory defect caused by the nature of their disease, but also because their spinal deformity impairs their height growth which further worsens their pulmonary function [6]. This study identified the most common complications during and after spinal surgical correction using pedicle screws of 14 NMS patients, who were followed for a two years period. The study highlighted some preoperative and postoperative patient-related characteristics that might have contributed to these complications.

In comparison to other types of scoliosis, NMS patients have a higher risk of blood loss during and after the operation [14]. Older age, lower BMI, a bigger number of fixed vertebrae, and a longer duration of surgery are all some of the predisposing factors for blood loss [14]. Thus, these risk factors must be identified before the operation to choose appropriate strategies for blood conservation in this population and optimize preparedness during and after the surgery. One study by Edler et al. that investigated if NMS patients were at higher risk of extensive blood loss during posterior spinal fusion surgery, concluded that they were seven times more likely to lose more than half of their estimated blood loss compared with non-NMS patients [15]. In this study, blood loss was the most commonly reported complication; 11 (85%) patients suffered from blood loss that required blood transfusion. Blood transfusions were given based on the acuity of blood loss during the operation. No clear preoperative risk factor was found in these 11 patients. Four had a normal BMI for their age, three were underweight for their age, and four were obese for their age. As for the age during surgery, the 11 patients’ ages were close ranging from 9 to 15 years; so older age was not an obvious predisposing factor for blood loss as reported by some studies [14]. However, it is expected in our sample since most of our patients had the correction from the level of T2 to pelvis which is an extensive amount of exposure requiring a large number of pedicle screws to be inserted [14]. Also, the nature of the underlying neuromuscular disorder itself makes it more challenging to insert these pedicle screws resulting in more blood loss. Finally, the duration of surgery in our sample ranged from 6 to 10 hours, which is a long time and carries a higher risk for blood loss [14].

The most common early postoperative complication reported was urinary tract infection (UTI) in two (15%) cases. All were treated with antibiotics after positive urine culture results. One study by Yousef and Rosenfeld which evaluated the causes of fever after surgical correction of NMS found that 25 (32.7%) of 76 patients had a fever and urinary tract infection was the most common cause of it [16]. Modi et al. who investigated postoperative complications amongst 50 NMS patients, reported UTI as a complication in eight (22%) patients and it was believed to be mainly due to urinary catheterization [17]. All the patients in our sample had a foley catheter placed for different durations, the shortest being one day and the longest being six days. The two patients in the sample who developed a UTI, both had their foley catheter removed on day three. This can be attributed to the use of foley catheters since it is a well-known risk factor for UTIs [18]. However, the longest period of foley catheter use in the study was six days, noted in three patients from the sample, none of whom developed a UTI. This supports that other unidentified causes might have contributed to the emergence of UTI in the two patients who developed it in our study.

Only two of the patients in the sample developed pulmonary complications which were pneumonia and atelectasis in the early postoperative period. Modi et al. found that 16 (43%) of their patients developed pulmonary complications [17]. Sarwahi et al. investigated the most prevalent major and minor complications among 111 NMS cases and found that the most common major complications in patients had been pulmonary [19]. As demonstrated by most of the literature, pulmonary problems were frequently reported as the most prevalent [9-12]. This is expected in this population due to their weaker respiratory function compared to the other types of scoliosis [6]. However, in our sample of patients, respiratory poor outcomes were much less prevalent compared with other outcomes such as blood loss. All of our patients were intubated during the procedure and most of them were extubated immediately after the surgery, which might have contributed to achieving better respiratory outcomes [20]. Also, some major postoperative measures, that were taken in our hospital, such as giving adequate analgesia and early involvement of a physiotherapist might have played a big role in preventing pulmonary poor outcomes [20]. Even though the sample size is not sufficient to compare our numbers with the literature, the low incidence of pulmonary complications in this study is promising and should be further investigated by future studies that cover a bigger sample size.

Other miscellaneous complications recorded in our study such as sepsis and wound infection had no clear predisposing factor. However, we did notice that they were often grouped with other complications such as UTI and pneumonia in the same patient. In the literature, wound infection was quite common among NMS patients [11,12]. The presence of ventriculoperitoneal shunt, history of seizures, history of previous operations, and presence of pulmonary comorbid conditions are all some of the significant predisposing factors for wound infection in patients with NMS [21,22]. Despite the long duration of the surgery and the heavy instrumentation used in all patients of the sample, only one developed a wound infection and did not have any history of chronic disease besides the neuromuscular disorder nor had a history of previous operations. The low incidence of wound infection in our sample might be attributed to the fact that all our patients had taken prophylactic antibiotics in the first 48 hours after surgery and none had comorbidities. None of the patients required reoperation for any of the documented complications. Nevertheless, future research should determine the associated risk factors with the complications of spinal surgical correction in the pediatric NMS population.

To name a few of the study’s limitations, the use of consecutive sampling and the small number of patients in the sample made it difficult to predict associated risk factors with these complications. Also, some patients did not have a two-year follow-up so they might have developed late postoperative complications that were not documented in their files.

## Conclusions

This is one of the very few studies that described the complications of scoliosis correction surgery in the pediatric NMS population in Saudi Arabia. The study concluded that blood loss was the most common intraoperative complication. The fact that no late postoperative complications were reported in our study is encouraging. Also, the low frequency of pulmonary complications in such a vulnerable group is hopeful for better outcomes in these children. 
